# Fluoxetine Prevents Aβ_1-42_-Induced Toxicity via a Paracrine Signaling Mediated by Transforming-Growth-Factor-β1

**DOI:** 10.3389/fphar.2016.00389

**Published:** 2016-10-25

**Authors:** Filippo Caraci, Fabio Tascedda, Sara Merlo, Cristina Benatti, Simona F. Spampinato, Antonio Munafò, Gian Marco Leggio, Ferdinando Nicoletti, Nicoletta Brunello, Filippo Drago, Maria Angela Sortino, Agata Copani

**Affiliations:** ^1^Department of Drug Sciences, University of CataniaCatania, Italy; ^2^Istituto di Ricovero e Cura a Carattere Scientifico Oasi Maria SantissimaTroina, Italy; ^3^Department of Life Sciences, University of Modena and Reggio EmiliaModena, Italy; ^4^Department of Biomedical and Biotechnological Sciences, University of CataniaCatania, Italy; ^5^Istituto di Ricovero e Cura a Carattere Scientifico NeuromedPozzilli, Italy; ^6^Department of Physiology and Pharmacology, University of Rome SapienzaRome, Italy; ^7^Institute of Biostructure and Bioimaging, National Research CouncilCatania, Italy

**Keywords:** Alzheimer’s disease, β-amyloid, neuroprotection, antidepressants, fluoxetine, cortical neurons, TGF-β1, depression

## Abstract

Selective reuptake inhibitors (SSRIs), such as fluoxetine and sertraline, increase circulating Transforming-Growth-Factor-β1 (TGF-β1) levels in depressed patients, and are currently studied for their neuroprotective properties in Alzheimer’s disease. TGF-β1 is an anti-inflammatory cytokine that exerts neuroprotective effects against β-amyloid (Aβ)-induced neurodegeneration. In the present work, the SSRI, fluoxetine, was tested for the ability to protect cortical neurons against 1 μM oligomeric Aβ_1-42_-induced toxicity. At therapeutic concentrations (100 nM–1 μM), fluoxetine significantly prevented Aβ-induced toxicity in mixed glia-neuronal cultures, but not in pure neuronal cultures. Though to a lesser extent, also sertraline was neuroprotective in mixed cultures, whereas serotonin (10 nM–10 μM) did not mimick fluoxetine effects. Glia-conditioned medium collected from astrocytes challenged with fluoxetine protected pure cortical neurons against Aβ toxicity. The effect was lost in the presence of a neutralizing antibody against TGF-β1 in the conditioned medium, or when the specific inhibitor of type-1 TGF-β1 receptor, SB431542, was added to pure neuronal cultures. Accordingly, a 24 h treatment of cortical astrocytes with fluoxetine promoted the release of active TGF-β1 in the culture media through the conversion of latent TGF-β1 to mature TGF-β1. Unlike fluoxetine, both serotonin and sertraline did not stimulate the astrocyte release of active TGF-β1. We conclude that fluoxetine is neuroprotective against Aβ toxicity *via* a paracrine signaling mediated by TGF-β1, which does not result from a simplistic SERT blockade.

## Introduction

Alzheimer’s disease (AD) is a neurodegenerative disorder characterized by memory loss, cognitive decline, and neuropsychiatric symptoms able to interfere with normal daily activities (Ballard et al., 2009). The ‘amyloid cascade hypothesis’ remains a widely accepted explanation of the etiology of AD also after recent revisions (Selkoe and Hardy, 2016). According to this hypothesis, the earliest event in the pathogenic cascade leading to dementia is the formation of aggregates of a 42-amino acid peptide called β-amyloid peptide (Aβ_1-42_). It is generally believed that oligomeric species of Aβ_1-42_ represent the key initiators of a complex pathogenic cascade that cause hyperphosphorylation of tau protein, synaptic dysfunction and finally neuronal death (Giacobini and Gold, 2013; Musiek and Holtzman, 2015). Soluble Aβ oligomers are highly toxic species that disrupt synaptic function and their burden correlates better with dementia severity than insoluble fibrillar deposits (Selkoe and Hardy, 2016). The neurotoxic effects of Aβ oligomers have been investigated *in vitro*, with different molecular mechanisms possibly explaining these effects, such as the amplification of NMDA toxicity (Hynd et al., 2004), the loss of the canonical Wnt signaling (Caricasole et al., 2004) and the activation of cell cycle in differentiated neurons (reviewed by Herrup et al., 2004). Neurotrophic factors, which seem to be deficient in the AD brain, including Brain-derived Neurotrophic Factor (BDNF) (Castren and Tanila, 2006), Nerve Growth Factor (NGF) (Iulita et al., 2016) and Transforming-Growth-Factor-β1 (TGF-β1) (Wyss-Coray, 2006; Caraci et al., 2011a), have been proposed to limit the neurotoxicity of Aβ oligomers.

TGF-β1 is a neurotrophic factor that exerts neuroprotective effects against β-amyloid-induced neurodegeneration (Caraci et al., 2008), and a selective impairment of TGF-β1 signaling pathway has been demonstrated in the early phase of AD pathogenesis (Tesseur et al., 2006). The +10 CC genotype of TGF-β1 gene, which affects the levels of expression of TGF-β1, increases the risk to develop Late-Onset AD, and is also associated with depressive symptoms in AD (>5-fold risk) (Caraci et al., 2012). Hence, a deficit of TGF-β1 seems to be a common pathophysiological event in both depression and AD (Caraci et al., 2010, 2014).

Depression is a risk factor for the development of AD, and the presence of depressive symptoms significantly increases the conversion of Mild Cognitive Impairment (MCI) into AD (Modrego and Ferrández, 2004). In animal models of amyloid -induced neurodegeneration Aβ_1-42_ can induce both a depressive state and memory deficits as observed in rodents (Colaianna et al., 2010; Morgese et al., 2014; Tucci et al., 2014). Interestingly, a continued long-term treatment with antidepressants reduces the risk to develop AD (Kessing et al., 2009). Among antidepressants, selective reuptake inhibitors (SSRIs), such as fluoxetine and sertraline, increase circulating TGF-β1 levels, which are reduced in major depressed patients (Lee and Kim, 2006; Sutcigil et al., 2007). Whether SSRIs cater the potential to be neuroprotective in AD, by rescuing TGF-β1 signaling, remains to be determined. Evidence exists that fluoxetine prevents amyloid pathology and reverses memory impairment in different animal models of AD (Wang et al., 2014; Jin et al., 2016). Here, we tested fluoxetine for its potential neuroprotective activity against Aβ toxicity and the prospective role of TGF-β1 in this phenomenon.

## Materials and Methods

### Handling of Aβ and Preparation of Human Aβ Oligomers

Synthetic human Aβ_1-42_ oligomers were prepared according to the original protocol of Klein’s group (Gong et al., 2003). Briefly, the Aβ_1-42_ lyophilized peptide, purchased from Novas Biologicals (Littleton, CO, USA), was dissolved in trifluoroacetic acid (TFA) (1 mg/ml) and sonicated in a water bath sonicator for 10 min. Then, TFA was evaporated under a gentle stream of argon, and 1 ml 1,1,1,3,3,3-hexafluoro-2-propanol (HFIP) was added to the peptide. After 1 h incubation at 37°C, the peptide solution was dried under a stream of argon, and then solubilized again by adding 2 ml of HFIP. Finally, HFIP was removed by argon streaming followed by further drying in a lyophilizer for 1 h, and Aβ_1-42_ then suspended in 5 mM anhydrous dimethyl sulfoxide (DMSO) before dilution to 100 μM in ice-cold cell culture medium DMEM-F12. Samples of Aβ_1-42_ at the concentration of 100 μM were incubated for 72 h at 4°C and then stored at -20°C until use. Aβ_1-42_ oligomers were used in neuronal cultures at a final concentration of 1 μM in the presence of the glutamate receptor antagonists MK-801 (10 μM) and DNQX (30 μM) to avoid the potentiation of endogenous glutamate toxicity.

### Drugs

Fluoxetine and sertraline were purchased from Sigma–Aldrich (St Louis, MO, USA). SB431542 and ARP-100 were purchased from Tocris (Bristol, UK). All the compounds were dissolved in DMSO at the initial concentration of 10 mM. The final concentration of DMSO applied to the cultures was 0.1%. Serotonin was purchased from Sigma–Aldrich (St Louis, MO, USA) and dissolved in PBS. The neutralizing antibody specific for TGF-β1 was purchased from R&D system (Minneapolis, MN, USA), was reconstituted in sterile PBS and used in neuronal cultures at a final concentrations of 2 μg/ml.

### Cultures of Cortical Neurons and Assessment of Neuronal Injury

Cultures of pure cortical neurons were obtained from rats at embryonic day 15 (Harlan Laboratories, Italy). Briefly, cortices were dissected in Ca^++^/Mg^++^ free buffer and mechanically dissociated. Cortical cells were plated at a density of 2 × 10^6^/dish on 35 mm dishes (Nunc, Rochester, NY, USA) pre-coated with 0.1 mg/ml poly-D-lysine (St Louis, MO, USA) in DMEM/Ham’s F12 (1:1) medium supplemented with the following components: 10 mg/ml bovine serum albumin, 10 μg/ml insulin, 100 μg/ml transferrin, 100 μM putrescine, 20 nM progesterone, 30 nM selenium, 2 mM glutamine, 6 mg/ml glucose, 100 U/ml penicillin, and 100 μg/ml streptomycin. Cytosine-D-arabinofuranoside (10 μM) was added to the cultures 18 h after plating to avoid the proliferation of non-neuronal elements and was kept for 3 days before medium replacement. This method yields >99% pure neuronal cultures, as judged by immunocytochemistry for glial fibrillary acidic protein and flow cytometry for neuron-specific microtubule-associated protein 2 (Copani et al., 1999).

For mixed cortical cultures, cortical cells were grown into DMEM/F12 (1:1) supplemented with 10% horse serum, 10% fetal calf serum (FCS), 2 mM glutamine, 6 mg/ml glucose. After 7–10 days *in vitro*, glia cell division was halted by exposure to 10 μM cytosine-D-arabinoside for 3 days and cells were shifted into a maintenance serum-free medium. Mature cultures contained about 35–40% neurons.

Neuronal cultures were treated at 7 days *in vitro* with Aβ_1-42_ oligomers (1 μM) for 48 h both in the presence and in the absence of fluoxetine (100 nM – 1 μM). Neuronal injury was assessed by the methyltetrazolium test (MTT) assay in pure neuronal cultures, and Trypan Blue staining in mixed neuronal cultures 48 h after Aβ_1-42_ treatment. For MTT assay cells were incubated with MTT (0.9 mg/ml final concentration, St Louis, MO, USA) for 2 h at 37°C. A solubilization solution containing 20% SDS was then added for an additional 1 h and formazan production was evaluated in a plate reader (λ = 560 nm). Aβ toxicity in mixed neuronal cultures was assessed by counting dead neurons stained with Trypan blue. Stained neurons were counted in three random microscopic fields/well.

## Pure Cultures of Cortical Astrocytes

Cortical glial cells were prepared from 1- to 3-day-old Sprague-Dawley rats. After removal of meninges and isolation of cortices, cells were dispersed by mechanical and enzymatic dissociation using a 0.25% solution of trypsin (Invitrogen). Cells were plated onto 75-mm^2^ flasks and maintained in DMEM, supplemented with 10% fetal calf serum, penicillin/streptomycin (100 U/ml–100 g/ml), and glutamine (2 mM). All medium constituents were from Invitrogen, and all plastic materials were from Corning Life Sciences (Lowell, MA, USA). Confluent cultures at 8–10 days *in vitro* were shaken overnight at 37°C to remove microglia and oligodendrocytes. Astrocytes were collected by trypsin digestion, seeded onto 35- or 100-mm dishes, and used for experiments 6–8 days after replating.

### Determination of TGF-β1 Levels in the Astrocyte Medium

Astrocyte-conditioned medium was collected and subjected to acid treatment procedure. Samples were acidified to a pH of approximately 2.6 with 1 N HCl for 15 min at room temperature, then neutralized to approximately pH 7.6 with 1 N NaOH. Levels of TGF-β1 released into the medium were measured by enzyme-linked immunosorbent assay using the TGFβ_1_ E_max_ Immunoassay System (Promega, Madison, WI, USA), based on an antibody sandwich format, strictly following the manufacturer’s instructions.

In brief, 96-well plates were coated overnight at 4°C with primary monoclonal anti-TGF-β1 antibody. A blocking solution was added for 35 min at 37°C before incubation with samples and standards for 90 min at room temperature, to allow binding of soluble TGF-β1. A primary polyclonal anti-TGF-β1 antibody was then added for 2 h to bind captured TGF-β1. Finally, specifically bound polyclonal antibody was detected by incubation for 2 h with a horseradish peroxidase-conjugated secondary antibody. Wells were extensively washed between each step. After a final 10-min incubation with achromogenic substrate solution, the resulting redox reaction was stopped by acidification with 1N HCl, and absorbance was immediately measured at 450 nm. The assay is sensitive in the range of 32–1000 pg/ml.

### Western Blot

Western blot analyses was performed as previously described (Caraci et al., 2015a) on neurons or astrocytes harvested at 4°C in RIPA buffer in the presence of a cocktail of protease inhibitors (Sigma–Aldrich P2714), serine/threonine phosphatase inhibitors (Sigma–Aldrich, P0044) and tyrosine protein phosphatases inhibitors (Sigma–Aldrich, P5726). Protein concentrations were determined by Bradford’s method using bovine serum albumin as a standard. After blocking, membranes were incubated with the following primary antibodies overnight at 4°C: rabbit anti-TGF-β1 (Abcam 25121, Cambridge, UK; 1:1000), rabbit anti-MMP2 (Santa Cruz Biotechnology, Santa, CA, USA; 1:500) and mouse anti-α-Tubulin and anti-β-Actin (Sigma–Aldrich; 1:500). Secondary goat anti-rabbit labeled with IRDye 680 (1:30.000 Li-COR Biosciences) and goat anti-mouse labeled with IRDye 800 (1:25.000 Li-COR Biosciences) were used at RT for 45 min. Hybridization signals were detected with the Odyssey Infrared Imaging System (LI-COR Biosciences). Western blot data were quantified by densitometric analysis of the hybridization signals in four different blots per experiment.

### Gene Expression Analysis by Real-Time RT-PCR

Total RNA was isolated from cultured astrocytes treated with fluoxetine (1 μM) using TRIzol reagent (Invitrogen), GenElute^TM^ Mammalian Total RNA Miniprep Kit and DNASE70-On-Column DNase I Digestion Set (St Louis, MO, USA) as previously described (Benatti et al., 2011). Two milligrams of total RNA was reverse transcribed with High Capacity cDNA Reverse Transcription Kit (Life Technologies Corporation, Carlsbad, CA, USA) in 20 μl of reaction mix. Real Time RT-PCR was performed in Roche Light Cycler^®^ 480 (Roche Diagnostics GmbH, Roche Applied Science, Mannheim, Germany) using Power UP SYBR Green mix (Life Technologies Corporation, Carlsbad, CA, USA). The following forward and reverse primers were used at the final concentration of 300 nM: TGF-β1 (NM_021578.2) Forward 5′-CCTTGCCCTCTACAACCAAC-3′, Reverse 5′-CTTGCGACCCACGTAGTAGAC-3′; Mmp-2 (NM_031054.2) Forward 5′-AGTTCTGGAGATACAATGAAG-3′, Reverse 5′-TCTCCAACTTCAGGTAATAAG-3′; glycera-ldehydes-3-phosphate dehydrogenase GAPDH (NM_017008.4) Forward 5′-CAAGGTCATCCATGACAACTTTG-3′, Reverse 5′-GGGCCATCCACAGTCTTCTG-3′. Single PCR products were subjected to a heat dissociation protocol as previously described (Alboni et al., 2013). Ct (cycle threshold) value was determined by the Light Cycler^®^ 480 Software (Roche Diagnostics GmbH, Roche Applied Science, Mannheim, Germany) mRNA expression was calculated with the ΔΔCt method with GAPDH as endogenous control. Two independent experiments were performed.

### Statistics

All experiments were blind with respect to treatment. Data were expressed as mean ± standard error mean (SEM). Statistical analysis was performed using dedicated software (GraphPad Prism, GraphPad Software). Data obtained from neuronal cultures and neuronal cell counts have been analyzed using a one-way ANOVA. The *post hoc* Bonferroni test was used for multiple comparisons; for western blot and gene expression analysis we used unpaired Student’s *t*-test; *p*-values <0.05 were considered as significant.

### Study Approval

The study was authorized by the Institutional Animal Care and Use Committee (IACUC) of the University of Catania (OPBA Project #169/2013). Animal care followed Italian (D.M.116192) and EEC (O.J. of E.C. L 358/1 12/18/1986) regulations on protection of animals used for experimental and scientific purposes.

## Results

**FIGURE 1:**
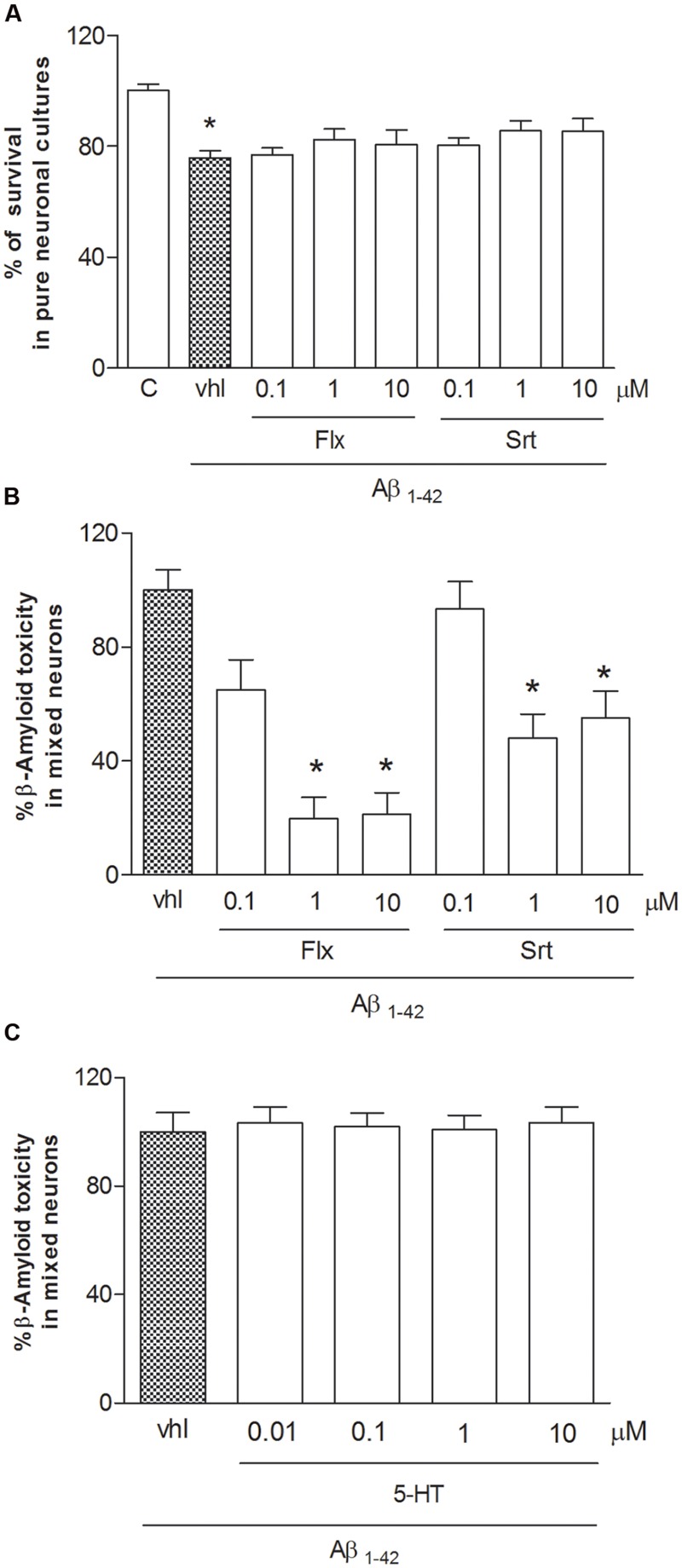
**Fluoxetine and sertraline prevent Aβ_1-42_-induced toxicity only in the presence of glial cells.** Antidepressant drugs were applied at increasing concentrations (100 nM–10 μM) both to pure **(A)** and mixed cortical neurons **(B)** in co-treatment with Aβ_1-42_ oligomers (1 μM) for 48 h. **(A)** Aβ toxicity in pure neuronal cultures is expressed as percentage of neuronal survival (quantified by MTT assay). Values in pure cortical neurons are means ± SEM of six to nine determinations ^∗^*p* < 0.05 vs. control **(C)** (One-way ANOVA + Bonferroni’s test). **(B)** Aβ toxicity in mixed neuronal cultures was assessed by cell counting after trypan blue staining. Cell count was performed in three random microscopic fields/well. Values in mixed cortical neurons are expressed as percentage of Aβ_1-42_ toxicity (vhl) and are means ± SEM of twelve determinations ^∗^*p* < 0.05 vs. Aβ alone (One-way ANOVA + Bonferroni’s test). **(C)** Serotonin (10 nM–10 μM) was co-applied with Aβ_1-42_ oligomers (1 μM) to mixed neuronal cultures for 48 h. The effects of serotonin against Aβ toxicity in mixed neuronal cultures were assessed by cell counting after trypan blue staining.

Both pure and mixed rat neuronal cultures were challenged with synthetic Aβ_1-42_ oligomers (1 μM) for 48 h. Because Aβ_1-42_ is able to potentiate glutamate toxicity, experiments were carried out in the presence of a cocktail of ionotropic glutamate receptor antagonists [MK-801 (1 μM) and DNQX (30 μM)] to exclude the contribution of endogenous excitotoxicity to the overall process of neuronal death. Under these conditions, neurons exposed to Aβ oligomers die showing an apoptotic phenotype (Copani et al., 1999; Caraci et al., 2015b). Exposure to Aβ_1-42_ oligomers (1 μM) was toxic in a time dependent manner, inducing apoptotic neuronal death in about 20–25% of neuronal population at 24 h, and 35–45% at 48 h. Fluoxetine or sertraline were co-applied with Aβ_1-42_ for 48 h at the concentrations (100 nM–10 μM) observed in depressed patients treated with these drugs. Fluoxetine (1–10 μM) prevented Aβ toxicity in mixed neuronal cultures (**Figure 1B**), but not in pure neuronal cultures (**Figure 1A**). Though to a lesser extent, also sertraline was neuroprotective in mixed cultures only (**Figure 1A**), suggesting that glial cells are essential to mediate the neuroprotective effects of both fluoxetine and sertraline. SSRIs, including fluoxetine and sertraline, block the serotonin transporter (SERT) that is located on the cell bodies and terminals of 5-HT neurons, as well as on cortical astrocytes (Inazu et al., 2001). Since SERT blockade results into an increased availability of serotonin, we tested serotonin (10 nM–10 μM) as a potential mediator of neuroprotection. As different from fluoxetine and sertraline, serotonin was devoid of effects (**Figure 1C**).

**FIGURE 2:**
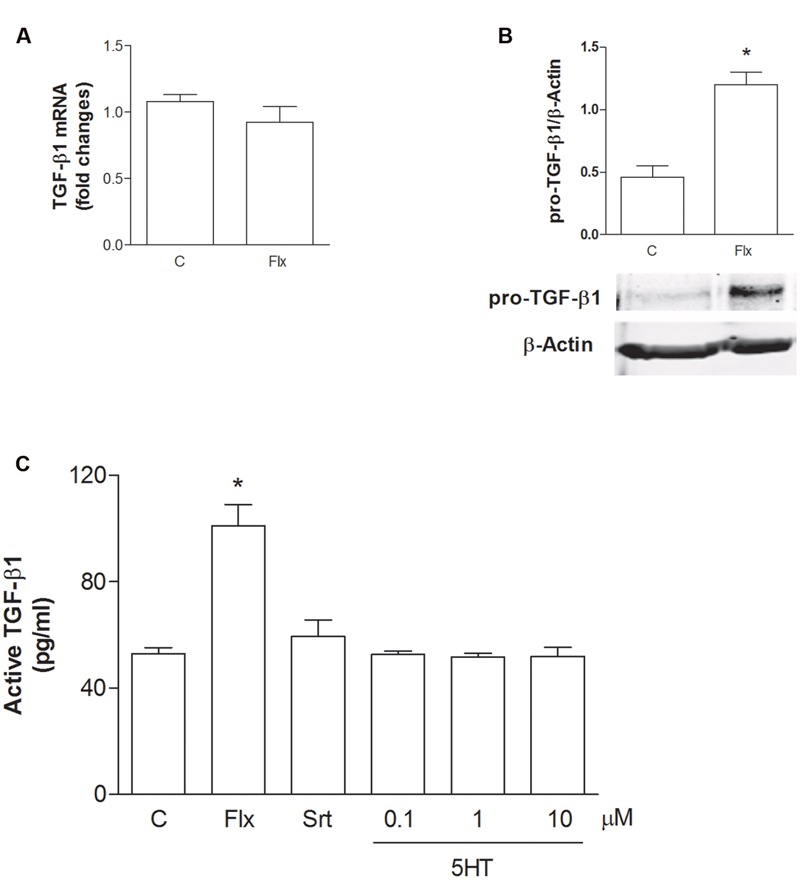
**Fluoxetine increases the expression of pro-TGF-β1 and the release of active TGF-β1 from cortical astrocytes. (A)** TGF-β1 mRNA levels obtained by Real-time RT-PCR in cultured astrocytes transiently exposed to fluoxetine (Flx; 1 μM) for 6 h are shown. Values were normalized by endogenous GAPDH mRNA levels and are represented as means + SEM for *n* = 4 for two independent experiments. **(B)** Representative immunoblots of pro-TGF-β1 (about 55 kDa) in total protein extracts from rat cortical astrocytes exposed to fluoxetine (Flx; 1 μM) for 24 h. Bars refer to the means ± SEM of the densitometric values of pro-TGF-β1 bands normalized against β-actin. Each experiment was repeated four times; ^∗^*p* < 0.05 vs. control by Student’s *t*-test. **(C)** Levels of active TGF-β1 in the medium of cultured astrocytes exposed for 24 h to fluoxetine (Flx; 1 μM), sertraline (Srt; 1 μM) or to increasing concentrations of serotonin (100 nM–10 μM) are shown. Values are means ± SEM of nine determinations; ^∗^*p* < 0.05 (Student’s *t*-test) versus untreated control astrocytes.

To understand the role of glial cells into the neuroprotective effects of SSRIs, we treated pure rat cortical astrocytes, the main source of TGF-β1 in CNS, with fluoxetine (1 μM) for 6 h or 24 h. In astrocytes, fluoxetine did not modify TGF-β1 mRNA levels at 6 h (**Figure 2A**), whereas it induced an increased synthesis of the precursor of TGF-β1 (Pro-TGF-β1) at 24 h (**Figure 2B**). In addition, we found significantly increased levels of active TGF-β1(101 pg/ml) in the medium of cultured astrocytes exposed to fluoxetine (1 μM) for 24 h with respect to untreated astrocytes (53,4 pg/ml) (**Figure 2C**). Neither sertraline nor serotonin stimulated the release of active TGF-β1 from cortical astrocytes (**Figure 2C**). These data suggested that, at least in the case of fluoxetine, the neuroprotective activity against Aβ could be mediated by astrocytes through the release of significant amounts of active TGF-β1.

**FIGURE 3:**
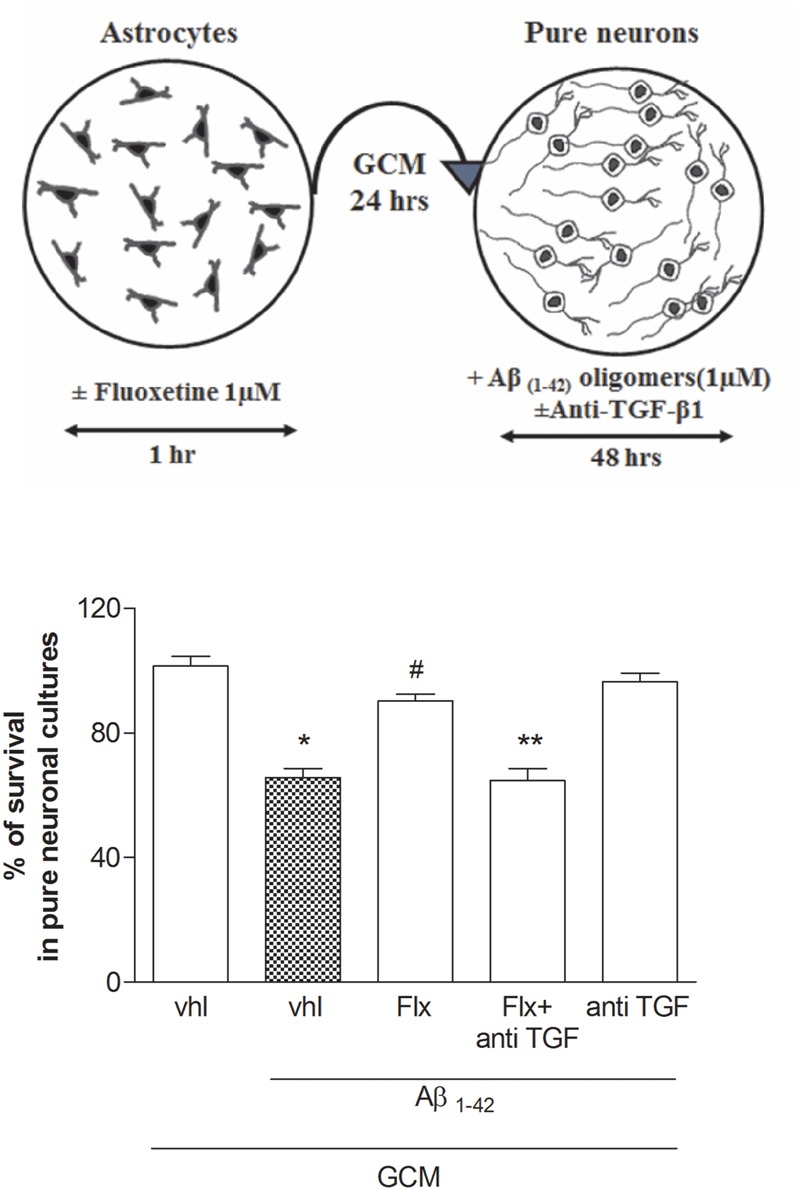
**Fluoxetine prevents Aβ toxicity via a paracrine mechanism mediated by TGF-β1.** Pure cultures of rat cortical neurons were exposed to glial conditioned medium (GCM) collected from cortical astrocytes 24 h after a transient (1 hr) exposure to1 μM fluoxetine or vehicle. Neurons were then treated with Aβ_1-42_ oligomers (1 μM) for 48 h in the presence or absence of anti-TGF-β1 antibody. A schematic drawing of this experimental protocol is shown in the upper panel. Anti-TGF-β1 was added at a concentration of 2 μg/ml just before transferring of GCM into pure neuronal cultures. Aβ toxicity in pure neuronal cultures was assessed by MTT assay and is expressed as percentage of neuronal survival. Values are means ± SEM of 12–15 determinations ^∗^*p* < 0.05 vs. control (GCM, vhl); ^#^*p* < 0.05 vs. Aβ_1-42_ alone (GCM, Aβ_1-42_, vhl); ^∗∗^*p* < 0.05 vs. Aβ_1-42_ and fluoxetine (GCM, Aβ_1-42_, Flx) (One-way ANOVA + Bonferroni’s test).

**FIGURE 4:**
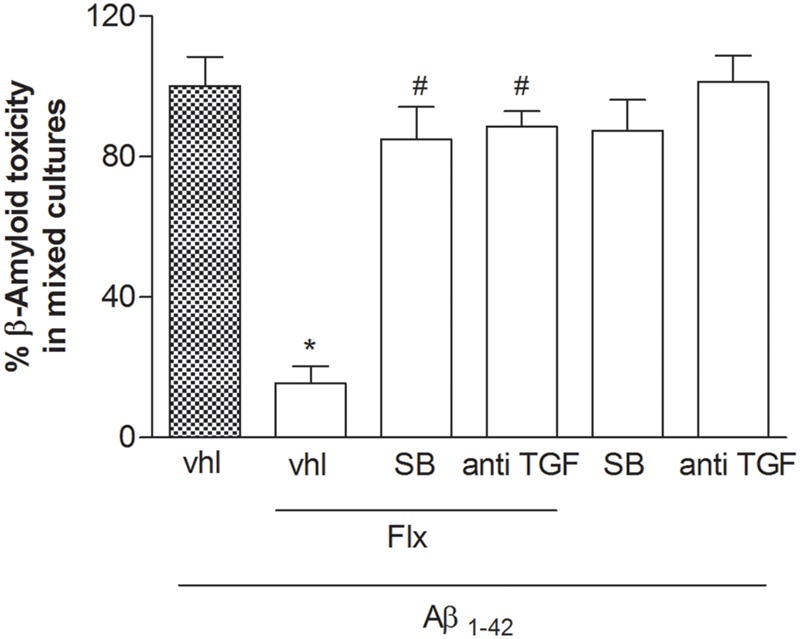
**The neuroprotective effects of fluoxetine against Aβ_1-42_-induced toxicity are mediated by TGF-β1.** Mixed cortical cultures were challenged with Aβ_1-42_ oligomers (1 μM) for 48 h in the absence or presence of fluoxetine (1 μM) applied alone or combined with the selective inhibitor of Smad-dependent TGF-β1 signaling, SB431542 (SB; 10 μM) or with a neutralizing antibody specific for TGF-β1 (anti-TGF-β1) applied at a concentration of 2 μg/ml. Aβ toxicity in mixed neuronal cultures was assessed by cell counting after trypan blue staining. Cell counts was performed in three random microscopic fields/well. Values are expressed as percentage of Aβ_1-42_ toxicity and are means ± SEM of nine determinations ^∗^*p* < 0.05 vs. Aβ alone (Aβ_1-42_, vhl) and #Aβ + fluoxetine (Aβ_1-42_, Flx, vhl) (One-way ANOVA + Bonferroni’s test).

To ascertain the contribution of astrocytes to the effect of fluoxetine, pure neuronal cultures were exposed to a glial conditioned medium (GCM) (i.e., medium collected from cultures of cortical astrocytes 24 h after a transient exposure to 1 μM fluoxetine or vehicle) before the treatment with Aβ_1-42_ oligomers. GCM collected from astrocytes challenged with fluoxetine protected pure cortical neurons against Aβ toxicity (**Figure 3**). A TGF-β1 neutralizing antibody (2 μg/ml), added to the astrocyte cultures, abolished the protective activity of the GCM derived from fluoxetine-treated astrocytes (**Figure 3**). Finally, both TGF-β1 antibodies and the specific inhibitor of type-1 TGF-β1 receptor, SB431542, (Laping et al., 2002) prevented the neuroprotective activity of fluoxetine directly applied to mixed cultures challenged with Aβ_1-42_ oligomers (**Figure 4**). SB431542 and anti-TGF-β1 had no effect on neuronal viability in the absence of Aβ_1-42_ (**Figure 4**). All together, these data demonstrate that the protective effect of fluoxetine on Aβ-treated neurons was mediated by an increased release of active TGF-β1 from cortical astrocytes.

**FIGURE 5:**
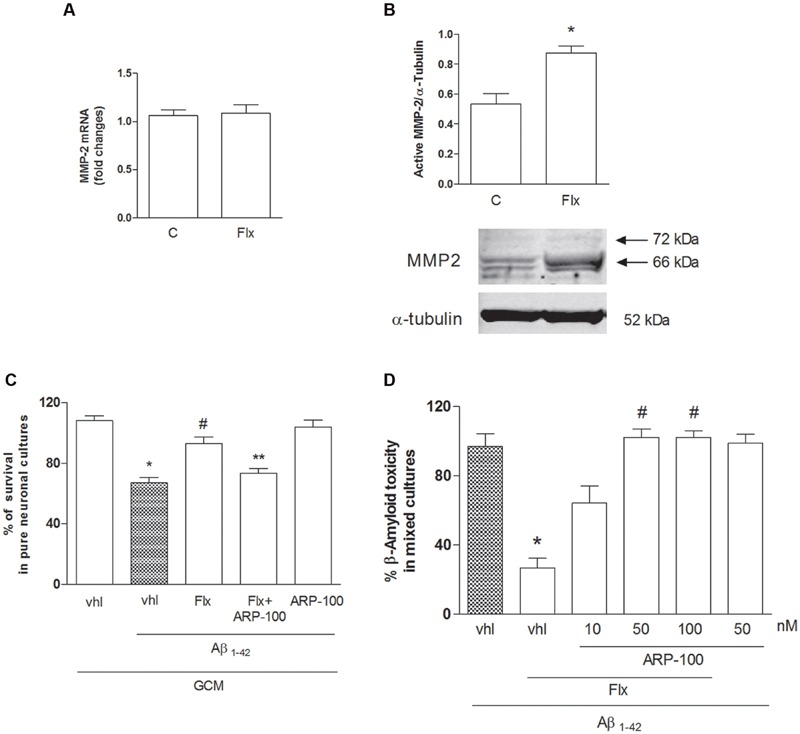
**Role of metalloproteinase-2 (MMP-2) in the neuroprotective effects of fluoxetine against Aβ toxicity. (A)** MMP-2 mRNA levels in cultured astrocytes transiently exposed to fluoxetine (Flx) for 6 h are shown. Values were normalized by endogenous GAPDH mRNA levels and are represented as means +SEM for *n* = 4 for two independent experiments. **(B)** Representative immunoblots of active MMP-2 in total protein extracts from rat cortical astrocytes exposed to fluoxetine (Flx, 1 μM) for 24 h. Arrows indicate pro-MMP2 (72 kDa) and the active form at about 66 kDa. Bars refer to the means ± SEM of the densitometric values of MMP-2 bands normalized against α-tubulin. Each experiment was repeated four times ^∗^*p* < 0.05 vs. control by Student’s *t*-test. **(C)** Pure cultures of rat cortical neurons were exposed to GCM collected from cortical astrocytes 24 h after a transient (1 hr) exposure to 1 μM fluoxetine with or without ARP-100 (50 nM), before treatment with Aβ_1-42_ oligomers (1 μM) for 48 h. Values are means ± SEM of 12–15 determinations ^∗^*p* < 0.05 vs. control values obtained with GCM alone (GCM, vhl), ^#^*p* < 0.05 vs. GCM+Aβ and ^∗∗^GCM+Aβ+FLX (One-way ANOVA + Bonferroni’s test). **(D)** Selective inhibition of MMP-2 prevents the neuroprotective effects of fluoxetine against Aβ toxicity. Mixed cortical cultures were challenged with Aβ_1-42_ oligomers (1 μM) for 48 h in the absence or presence of fluoxetine (1 μM) applied alone or combined with ARP100 (10–100 nM). Values are expressed as percentage of Aβ_1-42_ toxicity and are means ± SEM of twelve determinations ^∗^*p* < 0.05 vs. Aβ alone and #Aβ+fluoxetine (Aβ_1-42_, Flx, vhl) (One-way ANOVA +Bonferroni’s test).

TGF-β1 activity is primarily regulated through the conversion of latent TGF-β1 to active TGF-β1 by a variety of proteases (Annes et al., 2003), among which Matrix Metalloproteinase 2 (MMP-2), that is highly expressed in astrocytes (Li et al., 2011) and Matrix Metalloproteinase 9 (MMP-9) play a central role in this conversion (Jenkins, 2008). We examined the effects of fluoxetine on MMP-2 expression and activation in cortical astrocytes (**Figure 5**). Fluoxetine did not significantly modify MMP-2 mRNA levels at 6 h (**Figure 5A**), whereas it induced an increased expression of the active form of MMP-2 (66 kDa) at 24 h (**Figure 5B**). These data indicate that fluoxetine promoted the release of neuroprotective TGF-β1 by favoring the activation of MMP-2 and the ensuing maturation of latent TGF-β1. Accordingly, the GCM collected from fluoxetine-treated astrocytes in the presence of ARP-100, a selective inhibitor of MMP-2 at nanomolar concentrations (Tuccinardi et al., 2006), failed to protect pure cortical neurons against Aβ toxicity (**Figure 5C**). ARP-100 was also able to prevent the neuroprotective of fluoxetine when directly added to mixed cultured challenged with Aβ and it had no effect on neuronal viability in the absence of Aβ (**Figure 5D**).

## Discussion

Neuronal cultures challenged with synthetic analogs of human oligomers of Aβ_1-42_ are considered a widely accepted and reliable model of the neurodegeneration occurring in AD (Gong et al., 2003). In the present paper, synthetic human Aβ_1-42_ oligomers were prepared according to the original protocol of Klein’s group, as modified and characterized in Giuffrida et al. (2009). We then investigated the neuroprotective effects of two second-generation antidepressants in neuronal cultures challenged for 48 h with Aβ_1-42_ oligomers. We found that both fluoxetine and sertraline, two of the most currently prescribed SSRIs for the treatment of depression, prevented Aβ-induced neurodegeneration in cultures containing both neurons and glia, but not in pure neuronal cultures. Hence, glia cells mediated the neuroprotective effects of the two drugs.

Serotonin did not mimic the neuroprotective effects of fluoxetine and sertraline, suggesting that the increased serotonergic tone resulting from SERT blockade was not involved in the phenomenon.

Pharmacoepidemiological studies have demonstrated that a long-term treatment with SSRIs reduces the risk to develop AD in patients with depression (Kessing, 2012). A chronic treatment with SSRIs is associated with lower cortical β-amyloid PET signal in cognitively normal elderly human subjects (Cirrito et al., 2011), and with some degree of protection against the negative effects of depression on cognition in AD patients (Rozzini et al., 2010).

In different animal models of neuropsychiatric disorders, fluoxetine is known to induce the expression of neurotrophic factors, such as BDNF (Calabrese et al., 2009), insulin-like growth factor 1 (IGF-1) and glial cell line-derived neurotrophic factor (GDNF) (Allaman et al., 2011; Tizabi, 2016), whose source and potential neuroprotective effects remain unclear (Hashioka et al., 2013).

In the present study, we tested the hypothesis that fluoxetine and sertraline exert a neuroprotective activity against Aβ toxicity by stimulating the glia release of TGF-β1. TGF-β1 is known to prevent Aβ-induced neurodegeneration (Prehn et al., 1996; Caraci et al., 2008), and it acts as a master regulator of other neurotrophins (Sometani et al., 2001; Unsicker and Krieglstein, 2002; Schober et al., 2007). Surprisingly, fluoxetine, but not sertraline, increased the release of active TGF-β1 from cortical astrocytes. As in the case of other drugs able to induce TGF-β1 release from astrocytes, including estradiol (Sortino et al., 2004), and dual orthosteric agonists of metabotropic glutamate 2 (mGlu2) and mGlu3 receptors (Caraci et al., 2011b), we found that fluoxetine increased the intracellular levels of the precursor of TGF-β1 (Pro-TGFβ1) without affecting TGFβ1 mRNA expression. In addition, we demonstrate that fluoxetine affected the conversion of Pro-TGF-β1 into active TGF-β1, likely through the activation of MMP-2. The involvement of MMP-2 in fluoxetine activity is in line with the suggestion that extracellular matrix modifying enzymes contribute to antidepressant-mediated structural plasticity in the hippocampus (Benekareddy et al., 2008; Lee et al., 2014). The evidence that inhibiting astrocytes MMP-2 resulted into a lack of neuroprotection against Aβ toxicity supported the role of MMP-2 in fluoxetine-induced TGF-β1 release.

The reason why sertraline, which shares the same therapeutic target with fluoxetine (i.e., the SERT), did not induce astrocyte TGF-β1 release remains to be established. Further studies will be necessary to understand the molecular mechanisms underlying the neuroprotective effects of sertraline in the absence of TGF-β1 release, at least in our experimental model. We cannot exclude that sertraline could increase TGF-β1 levels *in vivo*, as observed in MMD patients, through the interplay between astrocytes and microglial cells (Hashioka et al., 2009).

Fluoxetine has been studied in different animal models of neurological disorders, including Parkinson’s disease, Down syndrome and ischemic brain injury (Zhu et al., 2012; Guidi et al., 2014; Caiaffo et al., 2016). Different molecular mechanisms have been proposed to explain its neuroprotective effects, including BDNF release (Chang et al., 2006), antagonism on NMDA receptors (Vizi et al., 2013), inhibition of NF-kappa B activity (Lim et al., 2009), and inhibition of the release of pro-inflammatory factors (TNF-α, IL-1β) from microglial cells (Zhang et al., 2012).

In the present work we identified a potential disease-modifying activity of fluoxetine depending on a paracrine mechanism mediated by TGFβ1. It is known that fluoxetine, during therapeutic treatment for major depression, accumulates in the brain up to a 20 μM concentration (Henry et al., 2005). Fluoxetine inhibits neuronal SERT with a *K*i value of 0.07 μM (Wong et al., 2005), whereas the brain concentration of fluoxetine is approximately 15–200 times higher than its binding affinity for SERT. Thus, fluoxetine may interact with additional pharmacological targets and act on non-neuronal cells to exert its clinical efficacy (Hashioka et al., 2013). We propose that stimulation of TGF-β1 release from astrocytes could represent an additional therapeutic mechanism for this antidepressant drug, which warrants further investigations in animal models of depression and AD. A similar neuroprotective mechanism may be postulated for venlafaxine, which, at least in astroglia-microglia co-cultures, stimulates TGF-β1-release (Vollmar et al., 2008).

Recent studies have demonstrated that a chronic treatment with fluoxetine prevents amyloid pathology and reverses memory deficits in two different animal models of AD (Wang et al., 2014; Jin et al., 2016). Based on the present finding, it would be relevant to assess whether fluoxetine can reduce AD-related pathology and prevent cognitive deficits by rescuing the TGF-β1 signaling. Interestingly, MCI patients treated with fluoxetine showed improvement in MMSE, and in immediate and delayed logical memory irrespective of the presence of depressive symptoms (Mowla et al., 2007). We have recently identified a key role for TGF-β1 in recognition memory formation, demonstrating that this neurotrophic factor is essential for the transition from early to late LTP (Caraci et al., 2015a). Deficit of TGF-β1 signaling is a primary event in AD pathogenesis and a reduced expression of type-2 TGF-β1 receptor specifically correlates with cognitive decline in early AD patients (Tesseur et al., 2006). Thus, the stimulation of TGF-β1 release from cortical astrocytes with fluoxetine might represent a novel pharmacological strategy to yield neuroprotection in AD.

## Author Contributions

FC gave substantial contributions to the conception and design of the work and approved the version to be published. FC, SM, CB, SS, and AM performed the experiments and approved the version to be published. FT and GL analyzed data and approved the version to be published. FT, FN, FD, and NB participated in the design of the study and approved the version to be published. FC, MS, and AC drafted the work and approved the version to be published.

## Conflict of Interest Statement

The authors declare that the research was conducted in the absence of any commercial or financial relationships that could be construed as a potential conflict of interest.
